# 5-Arylaminouracil Derivatives as Potential Dual-Action Agents

**Published:** 2015

**Authors:** E. S. Matyugina, M. S. Novikov, D. A. Babkov, V. T. Valuev-Elliston, C. Vanpouille, S. Zicari, A. Corona, E. Tramontano, L. B. Margolis, A. L. Khandazhinskaya, S. N. Kochetkov

**Affiliations:** Engelhardt Institute of Molecular Biology, Vavilova Str., 32, Moscow, 119991, Russia; Department of Pharmaceutical & Toxicological Chemistry, Volgograd State Medical University, Pavshikh Bortsov Sq., 1, Volgograd, 400131, Russia; Eunice Kennedy-Shriver National Institute of Child Health and Human Development, National Institutes of Health, Bethesda, MD 20892, USA; Department of Life and Environmental Sciences, University of Cagliari, Monserrato, 09042, Italy

**Keywords:** 5-(phenylamino)uracil derivatives, 5’-norcarbocyclic nucleoside analogs, HIV and Mycobacterium tuberculosis co-infection, dual action

## Abstract

Several 5-aminouracil derivatives that have previously been shown to inhibit *Mycobacterium tuberculosis* growth at concentrations of 5–40 μg/mL are demonstrated to act also as noncompetitive non-nucleoside inhibitors of HIV-1 reverse transcriptase without causing toxicity *in vitro *(MT-4 cells) and *ex vivo *(human tonsillar tissue).

## INTRODUCTION

Currently, HIV infection and TB are believed to be the major causes of infectious deaths worldwide. According to the latest WHO statistics, 9 million people were newly diagnosed with TB in 2013 and 1.5 million people died of TB (in 360,000 of these cases, TB was associated with HIV) [1]. In 2013, there were 35 million AIDS patients worldwide; 2.1 million cases of HIV infection were detected in 2013, and 1.5 million people died of AIDS, with TB remaining the main cause of death with dual infection (66.5%) [2]. HIV-infected patients are at an increased risk of latent tuberculosis reactivation (50% with TB vs. 10% without), and HIV-infected TB patients face a high risk of death. HIV patients receiving anti-TB drugs during a standard 6-month regimen are at a higher risk of recurrence than TB patients receiving a longer course of therapy [3]. Thus, TB and HIV co-infection is a very serious issue requiring a search for dual-action drugs.

**Fig. 1:**
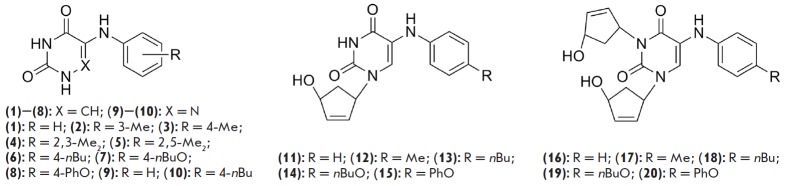


Recently, we demonstrated that some 5-arylaminouracil derivatives are capable of affecting active division of *Mycobacterium tuberculosis *cells. Total inhibition of mycobacterium growth by compounds (**2**), (**3**), (**6**), (**7**), (**10**), (**15**)**–(17**), and (**19**) (*Fig. 1*) was observed at concentrations of 5**–**40 μg/mL, with compound (**19**) exhibiting a higher activity against the MS-115 strain with multiple-drug resistance (including five major first-line anti-TB drugs: isoniazid, rifampicin, streptomycin, ethambutol, and pyrazinamide) than against the sensitive H37Rv laboratory strain [4].

This paper is devoted to the evaluation of 5-arylaminouracil derivatives as HIV NNRTIs and to a more detailed investigation of the toxicity of these compounds.

## EXPERIMENTAL

Compounds (**1**)–(**20**) were synthesized as previously described [4].

**1-(4’-Hydroxy-2’-cyclopenten-1’-yl)-3- benzyl-5-(phenylamino) uracil (21)**

K_2_CO_3_ (36 mg, 0.26 mM) and BnBr (42 μL, 0.35 mM) were added to a compound (**11**) solution (50 mg, 0.18 mM) in 5 mL of dimethylformamide (DMF). The reaction mixture was stirred at room temperature for 24 h. The reaction progress was monitored by means of TLC. We removed the solvent in an oil pump vacuum, purified the residue using column chromatography on silica gel, and eluted it with the system CHCl_3_–CH_3_OH (98:2). In total, 43 mg of the product (**21**) (yield of 66%) was obtained as a yellowish powder. R*_f_*= 0.32 (CHCl_3_– CH_3_OH, 98 : 2). ^1^H-NMR (CHCl_3_): 7.50–7.49 (2H, m, H_3_,H_5_-Bn), 7.32–7.23 (6H, m, H_2_”, H_3_”, H_5_”, H_6_”, H_2_,H_6_-Bn), 6.95–6.93 (2H, m, H_5_, H_4_-Bn), 6.90–6.88 (1H, t, H_4_”), 6.20– 6.18 (1H, m, H_2_’), 6.01 (1H, s, NH), 5.84–5.82 (1H, m, H_3_’), 5.61–5.58 (1H, m, H_1_’), 5.23–5.16 (2H, d, J = 13.70, CH_2_), 4.84–4.83 (1H, m, H_4_’), 2.86–2.85 (1H, m, H_a5_’), 1.70–1.66 (1H, m, H_b5_’). 13C-NMR (CHCl_3_): 160.80, 149.73 (C-4, C-2), 142.34 (C-4”), 139.24 (C-2’), 138.19 (C-4 Bn), 132.40 (C-3’), 129.63 (C-3”, C-5”), 129.34 (C-3, C-5 Bn), 128.63 (C-2”, C-6”), 127.91 (C-1”), 121.18 (C-1 Bn), 119.50 (C-5), 117.19 (C-6), 113.11 (C-2, C-6, Bn), 74.99 (C-1’), 61.05 (C- 4’), 45.49 (C-5’), 39.94 (CH_2_).

**1-(4’-Hydroxy-2’-cyclopenten-1’-yl)-3-benzyl- 5-(** *p* **-methylphenylamino) uracil(22)**

The synthesis was carried out as for (**21**), with (**12**) used as a starting compound. Of the product (**22**), 35 mg (yield of 68%) was obtained as a white-yellow powder. R*f*= 0.43 (CHCl_3_–CH_3_OH, 98 : 2). ^1^H-NMR (CHCl_3_): 7.50–7.48 (2H, m, H_3_,H_5_-Bn), 7.31–7.23 (4H, m, H2, H4, H_6_-Bn, H_5_), 7.06–7.04 (2H, m, H_3_”,H_5_”), 6.87–6.85 (2H, m, H_2_”, H_6_”), 6.18–6.16 (1H, m, H_2_’), 5.94 (1H, s, NH), 5.83–5.81 (1H, m, H_3_’), 5.58–5.56 (1H, m, H_1_’), 5.23–5.16 (2H, d, J = 13.76, CH_2_), 4.84–4.82 (1H, m, H_4_’), 2.87–2.83 (1H, m, H_a5_’), 2.26 (3H, s, CH_3_), 1.69–1.65 (1H, m, H_b5_’). 13C-NMR (CHCl_3_): 160.75, 149.66 (C-4, C-2), 139.57 (C- 4”), 139.14 (C-2’), 138.19 (C-4 Bn), 132.40 (C-3’), 130.14 (C-3”, C-5”), 129.33 (C-3, C-5, Bn), 128.67 (C-2”, C-6”), 127.88 (C-1”), 120.23 (C-1, Bn), 117.86 (C-2, C-6, Bn), 117.66 (C-5), 114.39 (C-6), 75.02 (C-1’), 61.12 (C-4’), 45.45 (C-5’), 39.94 (CH_2_), 20.76 (CH3).

**Anti-HIV activity**

Isolation of recombinant HIV-1 reverse transcriptase (p66/p51 heterodimer) and determination of its activity were performed as described earlier [5, 6]. The inhibition constant (*K_i_*), calculated according to Dixon’s method for noncompetitive inhibitors [7], was used as a quantitative measure of the inhibitory activity of the compounds. Nevirapine, a first-generation HIV NNRTI, was used as a control.

**Cytotoxicity in vitro**

We tested the compounds for potential cytotoxicity in the MT-4 cell line using an automatic cell counter (ChemoMetec). The number of live and dead cells was counted in the control cultures, and the cultures were treated with compound (**6**), (**7**), or (**19**). Compounds (**6**) and (**7**) were tested at concentrations of 0.136–33 μM (0.035–9 μg/mL), and compound (**19**) was tested at concentrations of 0.272–66 μM (0.119–28 μg/mL).

We discriminated live from dead cells by evaluating propidium iodide uptake according to the manufacturer’s instructions. We collected and analyzed data using the Nucleoview software (version 1.0,ChemoMetec).

**Toxicity *ex vivo***

The cytotoxicity of compounds (**6**), (**7**), and (**19**) was determined in human tonsillar tissues. A total of 27 tissue blocks were incubated with compound (**19**) (20 μg/mL) or with compound (**6**) or (**7**) **( **5 μg/mL) for each experimental point. Tissue blocks were cultured for 12 days. Then, the cells were isolated from the control and treated and stained with combinations of fluorescence- labeled antibodies against CD3-QD605, CD4- QD655, CD8-QD705, CD25-APC, CD38-PE, HLA-DRAPC- Cy7, CXCR4-Brilliant violet 421, CCR5-PR-Cy5 CD45RA-FITC, and CCR7-PE-Cy7 (Caltag Laboratories; Biolegend). We determined the numbers of cells of different phenotypes in isolated suspensions using flow cytometry as previously described. The volume of an analyzed suspension was controlled by means of Trucount beads (Becton Dickinson); the number of counted cells was normalized to the weight of the tissue fragments used for cell isolation.

## RESULTS AND DISCUSSION

The structural similarity of compounds (**1**)–(**20**) to uracil derivatives previously synthesized in our laboratory, together with their action as HIV NNRTIs [8, 9], suggested that these compounds might have similar properties. Compounds (**1**)–(**20**) belong to two groups: (**1**)– (**10**) are 5-arylaminouracil derivatives, while (**11**)–(**20**) contain one or two additional 4’-hydroxycyclopentene moieties and thus can be considered as 5’-norcarbocyclic analogs of 2’,3’-dideoxy-2’,3’-uridine. Despite the known structural similarity to nucleosides, the 5’-norcarbocyclic analogs are able to inhibit HIV reverse transcriptase through a non-competitive mechanism, binding at the so-called hydrophobic “non-nucleoside inhibitor binding center” [8, 9]. However, those among compounds (**1**)–(**20**) that inhibit the growth of *M. tuberculosis* did not possess the ability to inhibit HIV-1 reverse transcriptase (*K_i_* >> 200 μM). The only exception was compound (**15**) (*K_i_*= 119 μM), which belongs to the class of 5’-norcarbocyclic analogs of uridine.

**Fig. 2:**
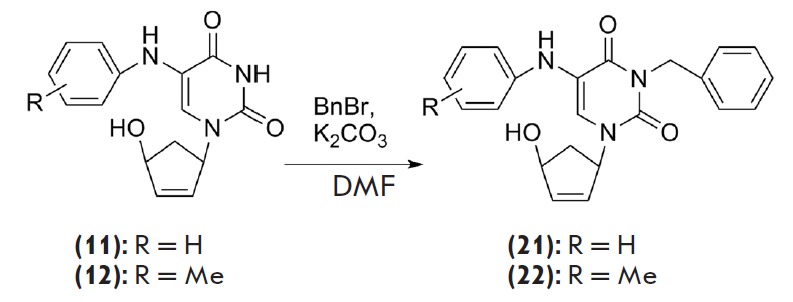


N^3^-benzyl derivatives (**21**) and (**22**) (*Fig. 2*) were synthesized to enhance the anti-HIV activity of compounds of this class by increasing their hydrophobicity. These compounds were obtained in acceptable yields (61–69%) through a reaction of the initial carbocyclic analogs (**11**) and (**12**) with benzyl bromide in the presence of potassiumcarbonate. We confirmed the structures and purity of the synthesized compounds using ^1^H- and ^13^C-NMR-spectroscopy and TLC. The inhibitory activity of the derivative (**22**) against HIV-1 reverse transcriptase appeared to be slightly higher than those of compound (**21**) (*K_i_*= 60 and > 100 μM, respectively) and the initial compounds (**11**) and (**12**).

Previously, we had assessed the cytotoxicity of the synthesized compounds on Vero, A549, and Huh7 cell lines and demonstrated that they were nontoxic at concentrations of up to 50 μg/mL (CD50 >> 100 μM). The toxicity of compounds (**6**), (**7**), and (**19**), which exhibited the most pronounced anti-TB properties, was further investigated *in vitro *on the MT-4 cell line and *ex vivo* on human tonsillar tissue.

Neither the cytotoxic nor the cytostatic effect of the compounds in MT-4 cells were observed at concentrations up to the maxima of 66 μM for (**19**) and 33 μM for (**6**) and (**7**).

Cytotoxicity assessment of the compounds (20 μg/mL for (**19**) and 5 μg/mL for (**6**) and (**7**)) on different cell types in the tissue system showed a lack of significant death rates of T cells (CD3+), B cells (CD3–), CD4+ and CD8+ T lymphocytes, and similarly for the CD4+ lymphocyte subgroups naive (CD45RA+/CCR7+), central memory cells (CD45RA–/CCR7+), effector memory cells CD45RA–/CCR7–), differentiated effector memory cells (Temra, CD45RA+/CCR7–), and activated CD4+ T-lymphocytes as well, with the last being identified as CD4+/CD25+, CD4+/CD38+T-cells, or CD4+/ HLA-DR+. In all of these groups, the number of cells in the control and treated tissues was the same.

Thus, despite the fact that the new 5-arylaminouracil derivatives showed no significant anti-HIV-activity, even the low activity of compounds (**15**) and (**22) **is indicative of their affinity for HIV-1 reverse transcriptase. The structural similarity of compounds of this type to many highly active antiviral agents of non-nucleoside nature which are used in HIV infection as components of complex, highly intensive antiretroviral therapy [10], in combination with their profound antituberculosis activity, makes them attractive targets for further modifications.

## Abbreviations

HIV: human immunodeficiency virus
TB: tuberculosis
WHO: World Health Organization
AIDS: acquired immunodeficiency syndrome
HIV NNRTIs: non-nucleoside inhibitors of HIV-1 reverse transcriptase
